# The effect of training medical students in the community area in the midst of the Covid-19 pandemic in China: a community-based study

**DOI:** 10.1186/s12909-023-04509-5

**Published:** 2023-07-18

**Authors:** Ying Li, YiYang Pan, XiWen Ding, Ayizuhere Aierken, Wei Jiang

**Affiliations:** 1 Department of Social Medicine, School of Public Health, Zhejiang University, Hangzhou, China; 2 School of medicine, Zhejiang University, Hangzhou, China; 3 College of Control Science and Engineering, Zhejiang University, Hangzhou, 310027 Zhejiang China

**Keywords:** Community practice training, Master of public health, Education, Community health, A quasi-experiment study

## Abstract

**Background:**

Community practice training is an important part of education in medicine, public health, social medicine, and other disciplines. The objective of this study is to explore the effect and importance of the community practice of Master of Public Health graduates on community residents’ health during the Coronavirus Disease 2019 pandemic.

**Methods:**

This study used a pretest-posttest design. A total of 152 participants with age ≥ 60 years were selected using a multistage sampling method from Hangzhou in China. Baseline and endline data were collected using structured questionnaires by face-to-face interviews. All psychological and behavioral measurements were performed using standardized instruments and showed good reliability and validity. A total of 147 participants were included in the analysis. The chi-square and rank sum tests were used to compare the difference between baseline and endline for categorical variables. Binary logistic regression analysis was used to evaluate the association between community practice training and changes in psychology and behavior.

**Results:**

The result of chi-square test revealed a statistically significant difference in participants’ eating habits from baseline to endline. Participants reported that the self-perceived health status was different between endline and baseline by the rank sum test. The results of logistic regression analysis showed that community practice training was significantly associated with increased self-efficacy scores, cognitive function and eating habits, with odd ratios of 1.08, 0.90 and 1.93, respectively.

**Conclusions:**

Community practice training was associated with changes in health behavior and psychology of community residents. Our results suggested enhanced community practice training for students under the Master of Public Health program.

**Supplementary Information:**

The online version contains supplementary material available at 10.1186/s12909-023-04509-5.

## Background

Community health service is the most extensive form of public health service and the pioneer field within the medical health system. Community health services cover a wide range of functions such as health education, prevention, health care, rehabilitation, and management of public health emergencies and play an important role in residents’ health. Community health education is the soul of community health service and one of the most important tasks of primary health care [1, 2].

Health education involves promoting people’s health and self-health management through various clinical, community, and other contexts by providing the necessary information and skills [3]. Previous studies have demonstrated that health education and health literacy have significant impacts at both the individual and community levels [4]. However, research on community health education during the COVID-19 pandemic is limited. In practical situations, there are often not enough personnel available to implement community health education effectively, or some forms of community health education may not have played a significant role in preventing the spread of COVID-19.

At the onset of the COVID-19 outbreak, Wuhan, China, had confirmed 46,201 cases by the end of February 2020 [5]. The Wuhan government implemented several prevention and control measures, such as closing the city, establishing designated hospitals, setting up shelters for centralized treatment, and isolating close contacts [6]. However, the lack of awareness of the epidemic among many community residents, especially the elderly, anxiety, illness concealment, and resistance to epidemiological investigation and epidemic management created difficulties in epidemic prevention and control [7–9]. Although China’s community health service is rapidly developing, public health professionals, especially community health service personnel, remain in short supply. In China, only 1.99 ‱ of community health service personnel account for public health physicians [10]. During the COVID-19 outbreak, an old community with a large number of elderly people had only 26 community staff, of whom only three were responsible for transporting fever patients to hospitals and for community disinfection and visitor registration. Each person was responsible for serving approximately 7,000 residents in their community [11].

In fact, 20 years ago, the Institute of Medicine called for investments in greater focus on public health education, particularly on a better link between public health education and practice [12–14]. The Public Health Accreditation Board was established to help guide education and public health training. The committee suggested increasing the availability of practice opportunities to develop students’ knowledge and skills, and the creation of real work environments of public health to promote problem-solving and competence development [15, 16]. The committee also recommended cooperation with the community to identify actual community health issues and build practice coursework around those issues to enhance students’ knowledge and skills. Briefly, community practice training is based on the community environment, focusing on social, behavioral, and cultural factors to better understand public health problems and identify comprehensive solutions to improve health, particularly in communities with poor public health conditions and underserved populations [17, 18]. This type of education will develop students’ skills and ethical competencies [19]. Community practice can promote students’ career intentions toward primary care and address enormous challenges caused by aging populations [20, 21]. The course content of practical training focuses on community health education and evidence-based approaches to public health, such as data collection, determination of health risk factors, statistical analysis, interpretation of quantitative and qualitative data, planning, and management. These techniques will promote community health assessment and diagnosis, intervention policy planning, and effect evaluation. It will also improve leadership skills, such as systems thinking, community participation, communication, advocacy and promotion, and teamwork [13].

According to the Ministry of Education of the People’s Republic of China in 2022, approximately 970,000 master’s degree students have academic degrees, accounting for 39.8% of the total number of master’s degree students [22]. The number of master’s degree students reached 1.474 million, accounting for 60.2% of the total number. For master of public health (MPH) training and the practice requirement for awarding degrees, most practice bases are set up in various medical and health institutions, such as centers for disease control and prevention, health education centers, and health supervision institutes [23, 24]. More than 90% of MPH students’ practical courses are conducted in the Centers for Disease Control and Prevention, and only 1.9% are completed in community health service institutions [25, 26]. The community is rarely considered as a public health practice base, and the community MPH education system has not been established. Community health education is an important part of public health education and provides an effective way to improve community residents’ health knowledge and literacy. Interactive sessions can lead to good engagement of health education and good acquisition of health knowledge. In China, community health education is often promoted through the distribution of posters or leaflets in the community [27, 28]. However, this form of community health education may not be effective for some elderly individuals who experience difficulties in reading and understanding the material. Moreover, some elderly individuals may not be able to read at all. Given that older adults have been identified as a particularly vulnerable group during the COVID-19 pandemic, it is important to ensure that community health education efforts are tailored to meet the specific needs of this population. Prevention and management strategies, such as social distancing, may increase the risk of social isolation and loneliness among older adults, further underscoring the need for targeted community health education efforts [29–31].

We hypothesize that community-based practical education can enhance the health knowledge acquisition of community residents. Therefore, this study aimed to investigate the impact of an MPH student’s community training course based on the theoretical framework of practice for the health of elderly individuals residing in the community during the COVID-19 pandemic [32].

## Methods

### Study design and participants

A community-based study was designed to investigate the effect of community practice training on the health of the elderly who live in the community. This is a quasi-experiment with pre-test and post-test design. On the basis of empirical study, 152 community residents with age ≥ 60 years were selected from Hangzhou, Zhejiang Province in China during the period of September 2020 to July 2021. The multi-stage sampling was conducted to determine the three communities by the students by using random method from three subdistricts of West Lake district. First, from all districts in Hangzhou, West Lake was randomly selected. Then, three subdistricts in West Lake were randomly selected from all West Lake subdistricts. Finally, three communities with good organizational and managerial capacities were selected from the three aforementioned subdistricts. The size of the selected communities and villages were relatively small at about 2,500–5,000 permanent residents. Participants were enrolled by community staff members through phone calls.

The practical training lasted for a week. The training lasted approximately 150 h in the pre-test in September 2020, and in the post -test in July 2021, respectively. Each participant took approximately 60 min to complete each interview. The practical training team consisted of nine students under the Master of Public Health program. In the three communities, the investigators were the same MPH students. The purpose of this study was explained by the research team, and all participants provided written informed consent before participation in this study. If participants were illiterate that the oral informed consents were provided. The study and method of oral informed consent were approved by the Institutional Review Board of the School of Medicine, Zhejiang University (No: ZGL201909-10).

### Practical training setting

This method aims to allow the students to complete practical training contents, such as sampling, data collection, identification of risk factors, statistical analysis, data interpretation, planning, and management to promote health, community diagnosis, effect evaluation of intervention, and leadership skills, community participation, communication, advocacy and promotion, and teamwork. Before the formal survey and during the project meeting, the multi-stage sampling was conducted to determine three communities by the students by using random lottery method. After determining the number of participants in each community, the students called the community staff, explained the research content and purpose, obtained agreement and cooperation, showed their identity and organization certificates, and determined the survey date and the method recruit participants by telephone with community managers. Students conducted in-depth interviews with community staffs to obtain the general information about the community before the survey. Semi-structured interviews and health communication and advocacy promotion technology were applied to this survey. The practical training spanned for a week, during which the practice training team visited three different communities. On the day of the survey, community staff called the next participant as the previous participant started the survey. These participants were allocated to each investigator once each investigation ended. The main activity during the community practice training was health education, which involved five steps.

Step 1: Identifying the problem. The interviewer asked the participants what they knew about COVID-19 and how it spreads.

Step 2: Communicating health knowledge. The interviewer interpreted COVID-19, its mode of transmission, and main symptoms to the participants.

Step 3: Promoting health behavior. The participants received self-care knowledge from the interviewer, such as diet, sleep, and well-ventilated rooms.

Step 4: Assessing health needs. The interviewer asked the participants whether they or their family members have ever been infected with COVID-19 and whether they are afraid and in need of help.

Step 5: Health education and problem-solving. The interviewer introduced community resources and provided contact information in case of an emergency to help participants feel supported.

Overall, these five steps comprised the health education activities that took place during the community practice training. Nine months after the completion of the training program, we assessed the participants’ cognitive function and related mental health indicators.

### Data collection

A structured questionnaire was used to investigate the elderly living in the community. The questionnaire was divided into nine parts and comprised 520 items. Before the investigation, we conducted a preliminary investigation to test the validity of the questionnaire. First, 30 elderly people who met the inclusion criteria were selected from other communities. A face-to-face preliminary investigation was conducted using the initial questionnaire. After the investigation, the participants were asked whether they understood all the contents of the questionnaire, their opinions on the items were recorded, and their understanding for the items was evaluated by the investigator. In the preliminary investigation, all subjects clearly understood the items, and the effective response rate was 100%. Next, confirmatory factor analysis was used to analyze the validity of the questionnaire, and the value of Kaiser-Meyer-Olkin was 0.889.

The main content of the questionnaire comprised demographic characteristics, behavior and habits, neurological and social cognition, personality and psychology, community resources, health and perceived health status, social support, adverse events and environmental factors, and community services during the COVID-19 pandemic. The demographic characteristics of participants included age, gender, marital status, income, and education level. Daily behavioral habits were asked from the following aspects: eating habits, tobacco use, alcohol consumption, sleep duration and quality, and physical activity. Data on the subjects’ eating habits were collected using the item “Do you eat regularly every day?” The answers were divided into “Very regular,” “Regular,” “Not very regular,” or “Irregular.” The participants were also asked “How many meals do you normally eat every day?” “How often do you eat snacks or fruits every day?” and “Do you drink tea?” Regarding behavioral habits, the participants were also asked the following items: “Do you smoke?” “Do you drink? “Do you regularly participate in physical activity?” and so on. If the responses were “Yes,” they were then asked about the specific kind and frequency. Personality and psychological factors were evaluated in accordance with the following: self-efficacy, depression, dependency, and personality. Community resources were divided into four dimensions: environmental, medical, nursing, and welfare resources. Self-reported chronic disease status and self-perceived health level were recorded. All data were collected at baseline and end of observation. Face-to-face structured interviews were conducted by Master of Public Health students who used a standardized interview protocol and a set of responses for recording the responses of participants. The interview time was approximately 45–60 min for most participants, and the interviewer suggested that elderly people who feel tired may take a rest [33].

### Measurements

Self-efficacy was measured using the Chinese version of General Self-Efficacy Scale (GSES). The GSES consists of 10 items, and 10–40 points may be obtained [34]. High scores represented high levels of self-efficacy and the Cronbach’s alpha coefcient (Cronbach’s α) was 0.89 for total scale. Cognitive function was assessed using the Chinese version of Dementia Assessment Sheet for Community-based Integrated Care System 21 items (DASC-21) with a Cronbach’s α of 0.86 [35]. The total score was 84 points, and a high score indicated poor cognitive function. The 15-item Geriatric Depression Scale was used to identify individuals with depressive symptoms [36]. The cut-off point to determine depressive symptoms was 5 points, and the Cronbach’s α was 0.75. Dependency personality disorder was identified using the Chinese version of standardized Minnesota Multiphasic Personality Inventory-II [37]. Dependency was identifed when individuals obtain a standardized score greater than or equal to 60 points. The test–retest reliability was 0.81 in women and 0.67 in man, and the criterion-related validity was − 0.70. Social support was assessed using the Older American Resources and Services scale [38]. Items are summed to yield a total score, with high scores indicate high levels of social support. The Cronbach’s α ranged from 0.61 to 0.83. Personality were identifed using the Chinese version of Eysenck Personality Inventory (EPI) [39]. The short EPI consists of 48 items and high scores indicate personality of extraversion, neuroticism, and psychoticism. The test–retest reliability was 0.74 to 0.77 for three dimensions. All measurement instruments used in the study showed good reliability and validity.

### Statistical analysis

Among participants, one participant was hospitalized, and four participants went to their children’s homes at endline. Statistical analysis was restricted to the 147 participants with complete questionnaires and follow-up survey data. Frequency and percentage were used to describe the general characteristics of the study participants. Chi-square and rank sum tests were used to analyze categorical variables. Baseline and endline pairwise comparisons were performed post-hoc to investigate specific relationships within those analyses. Continuous variables were compared using t-test or Mann–Whitney test. A logistic regression analysis was conducted to identify the association between community practice training and changes in psychology and behavior of participants. Self-efficacy, DASC-21, and dependency scores were divided into “1” and “0” by using the 75th percentile. Outcome variables to be observed were added to the logistic regression model as binary dependent variables. Community practice training was defined as two categorical variables, which were assigned a value of “0” at baseline survey and “1” at the end of observation, and community practice training and other factors were added to the model as independent variables. Reverse validation was performed using continuous variables. The significance level for all analyses was set at P < 0.05. All analyses were performed using SAS for Windows (version 9.4).

## Results

The baseline characteristics of the participants are shown in Table 1. Among all the participants, 105 (71.4%) were female, and 42 (28.6%) were male. About 45.6% participants were at least 70 years old, and 78.9% of participants reported one or more chronic diseases. Approximately 68.7% of participants reported lower level of education and completed only nine years or less of education. A high proportion of participants (86.4%) also reported physical activity during the COVID-19 pandemic.

**Table 1:** Baseline characteristics of study participants

Characteristic variables	n	%
Gender		
Male	42	28.6
Female	105	71.4
Age (yr.)		
< 70	80	54.4
≥70	67	45.6
Education levels (yr.)		
0–6	18	12.2
7–9	83	56.5
10–12	38	25.9
12+	8	5.4
Marital status		
Married	128	87.1
Non-married	19	12.9
Individual income		
¥0 to 3,999	123	83.67
¥4,000 and over	24	16.33
Smoking status		
Yes	20	13.6
No	127	86.4
Alcohol use		
Yes	48	32.6
No	99	67.4
Physical activity		
Yes	127	86.4
No	20	13.6
Chronic disease status		
Yes	116	78.9
No	31	21.1

Table 2 shows the changes in behavior and psychology after community practical training. About 37.4% and 21.1% of the participants reported irregular eating habits at baseline and endline, respectively (p < 0.001). About 10.2% and 19.7% of participants were light drinkers at baseline and endline, respectively. Light drinkers were significantly increased in the endline. By contrast, about 21.8% and 17.0% of participants were moderate to heavy drinkers at baseline and endline, respectively (p = 0.059).

**Table 2:** Changes in behavior and psychological after community practical training

Category Variables	Baseline survey		Endline survey		
	n	%	n	%	*P*
Alcohol use					
No drinking	100	68.0	93	63.3	0.059
Light drinking	15	10.2	29	19.7	
Moderate-heavy drinking	32	21.8	25	17.0	
Eating habits					
Very regular	92	62.6	116	78.9	0.001
Not very regular	55	37.4	31	21.1	
Self-perceived health					
Very good	11	7.5	22	15.0	0.036
Good	62	42.2	63	42.8	
Ordinary	56	38.1	55	37.4	
Not good	18	12.2	7	4.8	
Continuous variables (Mean, SD)					
Self-efficacy scores	25.7	6.1	29.0	6.7	< 0.001
DASC-21 scores	26.3	3.4	25.0	3.1	0.001
DYS scores	40.5	9.4	38.5	9.9	0.079
Depression scores	1.9	2.5	2.2	2.1	0.300
Time of physical activity	51.6	25.6	56.5	35.0	0.203

DASC-21: Dementia Assessment Sheet for Community-based Integrated Care System 21-items

DYS: Dependency scores

In terms of self-perceived health status, 7.5% and 12.2% participants reported “very good” and “not good”, respectively, at baseline, and these values increased to 15.5% and decreased to 4.8%, respectively, at endline (p < 0.05). Participants had significantly higher self-efficacy scores in the endline survey than in the baseline survey (p < 0.001). By contrast, the DASC-21 scores of participants were 26.3 and 25.0 points at baseline and endline, respectively (p < 0.05). Dementia scores measured by DASC-21 significantly decreased, indicating an improvement in cognitive function among participants. More detailed results can be found in Supplemental Table 1.

The association of community practice training with changes in psychology and behavior analyzed by logistic regression analysis is shown in Table 3. Community practice training was significantly associated with increased self-efficacy and decreased DASC-21 scores, with ORs of 1.08 (95% CI = 1.04–1.13, p < 0.001) and 0.90 (95% CI = 0.83–0.98, p < 0.05), respectively. Community practice training was associated with altered eating habits from irregular to very regular, with OR of 1.93 (95% CI = 1.18–3.15, p < 0.01). The association between community practice training and self-perceived health status no longer maintained a significant level after adjustment for gender, education levels, marital status, physical activity, individual income, and social support.

**Table 3:** The association between community practice training and change in health behavior and psychology by logistic regression analysis

Variables	Multivariable adjusted			*P*
	Odds Ratios	95% CI		
Self-efficacy scores (points)	1.08	1.04	1.13	< 0.001
DASC-21 scores (points)	0.90	0.83	0.98	< 0.013
Eating habits (irregular to very regular)	1.93	1.18	3.15	0.009
Age (yr)	1.08	1.03	1.13	0.001
SPH (not good to very good)	1.19	0.86	1.66	0.285
DYS scores (points)	1.01	0.98	1.04	0.495

Model adjusted for gender, education levels, marital status, physical activity, individual income and social support

SPH: self-perceived health

DASC-21: Dementia Assessment Sheet for Community-based Integrated Care System 21-items

DYS: Dependency scores

## Discussion

In this study, community practice training is significantly associated with changes in behavior and psychology among the elderly people living in a community during the COVID-19 pandemic. After the community practice training, participants have high self-efficacy and cognitive levels compared with the situation before community practice training.

It is now widely recognized that the most effective approach to promoting health and reducing health inequalities is to create more equal political, economic, social, and educational conditions. For over 50 years, popular education has been successfully used to promote more equal health education conditions around the world [40]. Several epidemiological studies have demonstrated the considerable impact of community health education on primary healthcare and clinical disease outcomes. For example, community health education can reduce the prevalence of risk factors for various chronic diseases at the community level and the morbidity and mortality associated with cardiovascular diseases, AIDS, diabetes, and other chronic diseases. However, as we have mentioned in the previous part, the lack of research on community health education and necessary resources for implementing effective health education due to the COVID-19 pandemic has led to the absence of reliable evidence on how to implement community health education and assess the impact of community health education on community residents’ health during epidemics. In this present study, we observed that community health education can effectively improve the health behavior, cognition, and self-efficacy of the elderly living in the community during the COVID-19 pandemic. To our knowledge, there are few studies that examine the conditions of community health education for elderly individuals during the COVID-19 pandemic. We believe that our study contributes to this important area of research.

The COVID-19 pandemic has brought great changes to everyone’s daily life and environment, especially the elderly people. The elderly people are considered to be the most vulnerable and affected group. They have the highest risk of infection with COVID-19 and death [41]. Some COVID-19 prevention and control measures have changed their lifestyle dramatically, seriously interfering with their access to health resources. Considering that they are left unattended at home, they could not obtain any information about the epidemic and related health knowledge. The prevention and control measures of “clearing the social dimension” have not been recognized by the elderly people as a protective measure. Instead, it has increased the anxiety, helplessness, and fear of the elderly people [23, 42, 43]. They pay more attention to whether anyone around them is taken to the “isolation point” or declared dead. The implementation of the isolation policy not only affects their access to health knowledge and social support, but also brings great harm to the physical and mental health of the elderly people [26]. In addition, in the present study, 71.4% of the participants are female. Our previous research and other studies have shown that women are more actively involved in community and health-promotion activities than men are because women are more innovative than men late in their life; they tend to be more receptive to new leisure activities and engage at a high frequency [44, 45].

During the COVID-19 pandemic, the inequality of health education was prominent, and the elderly people who originally lacked educational resources maintained the same status. This community practice training provides these elderly people with a good opportunity to receive health education [46, 47]. The student interviewer strongly felt the excitement and the reluctance to end the interview among the elderly people who participated in the investigation. This community practical training provides a good opportunity to receive health education for these elderly people. Self-perceived health and eating habits were remarkably improved, while self-efficacy and cognitive levels substantially increased among the elderly in the endline survey. The convergence was observed possibly because long-term environmental exposure can lead to the body and cognition function to adapt gradually to environmental changes, and the role of health education based on community practice training can be recognized. This result is also confirmed in our further analysis, and the changes in self-efficacy, cognitive level, and eating habits at endline are significantly associated with community practice training.

The outbreak of the COVID-19 pandemic resulted in great threats to the health of individuals and families and profoundly affected the entire medical and educational system. The COVID-19 pandemic has changed the global education model for hundreds of years. It is manifested by the shift from face-to-face teaching to home-based learning in college, middle school, and primary school during the COVID-19 pandemic. Some learning methods such as massive open online courses, YouTube, Twitter, and Mobile Apps have been developed. During the COVID-19 pandemic, many students have transitioned their face-to-face learning to online learning based on video-based platforms. Community environment and health assessment [18, 48], project planning, survey implementation and evaluation, and direct community practice are the core contents of community public health practice education [49, 50]. Community practice training is also the basis for all public health research, intervention strategy formulation, public health policy implementation, and disaster prevention [51, 52]. Applied investigation is also an effective way of health education and health promotion and covers the main contents of practical education and training of community public health [53, 54]. The period of major public health events and disease epidemics is the best time for community diagnosis and implementation of public health projects. These projects can quickly identify public health problems, implement emergency preventive strategy, guide health promotion, and enhance the community comprehensive prevention and control capacity. In this study, elderly people were selected as study subjects because of the high risk of disease and the remarkable effect on the daily health behavior and mental health of this age group. Changes in the behavior and psychology of these elderly people is also easy to observe at endline. The COVID-19 pandemic provides a good opportunity to obtain professional knowledge and practical skills for MPH students. After the community practice training, the students observed that the psychology and behavior of study participants had a healthy outcome, thus strengthening their determination and beliefs of participating in community public health work [55, 56].

The main strength of this study is that it is a pretest–posttest design. It is a useful way of ensuring that an experiment has a strong level of internal validity. In addition, a large number of data was obtained, allowing the analysis of many health-related factors. This study also has several limitations. First, this study has a relatively small sample size. Although the study is a good community practice training and education project, the COVID-19 pandemic and the change in implementation policy will lead to changes in the outcome of some studies, such as cognitive function, which needs to be verified in similar studies. Based on the theory of behavior change, we determined that the interval between the pre-test and post-test assessments would be six months. However, due to changes in community access management during the COVID-19 pandemic, we conducted the post-test nine months after the pre-test. As a result, this may introduce additional confounding and bias into the study. Moreover, the results are difficult to extrapolate to all people. In addition, considering the pandemic, not many health promotion plans and interventions were implemented. Our study suggests that public health education during the pandemic of major diseases should not only develop online learning but also carry out community practice training. Community practice training is a good community practice course for increasing training skills of MPH students, and it can promote the health of community residents.

## Conclusions

In conclusion, this study highlights the potential of community practice training as a means of promoting the health of community residents, particularly the elderly and other vulnerable groups, during major disease outbreaks such as the COVID-19 pandemic. Our findings underscore the importance of community health education as a critical component of community practice training. As such, we recommend that the skills of community health education be further strengthened for MPH students to better equip them to promote public health in community settings.

## Electronic supplementary material

Below is the link to the electronic supplementary material.

Supplementary Material 1

## Data Availability

The datasets generated and analysed during the current study are not publicly available due to ensure the privacy of participants but are available from the corresponding author on reasonable request.

## Abbreviations

MPH: Master of Public Health
COVID-19: Corona Virus Disease 2019
GSES: General Self-Efficacy Scale
DASC-21: Dementia Assessment Sheet for Community-based Integrated Care System-21 items
EPQ: Eysenck Personality Questionnaire
ORs: Odds ratios
CI: Confidence interval
