# Supplementary material for: The effect of training medical students in the community area in the midst of the Covid-19 pandemic in China: a community-based study
Source: BMC Med Educ. 2023 Jul 18;23:517. doi: 10.1186/s12909-023-04509-5 (PMC10354964; doi:10.1186/s12909-023-04509-5)
Supplement: Supplementary file 1 — Supplementary Material 1 [file 12909_2023_4509_MOESM1_ESM.doc]

**Supplemental table 1.** **Changes in behavior and psychological after community practical training by pairwise analyses**

| Category Variables | Baseline survey | | Endline survey | |  |
| --- | --- | --- | --- | --- | --- |
|  | n | % | n | % | *P* |
| Alcohol use |  |  |  |  |  |
| No drinking | 100 | 68.0 | 93 | 63.3 | 0.390 |
| Light drinking | 15 | 10.2 | 29 | 19.7 | 0.022 |
| Moderate-heavy drinking | 32 | 21.8 | 25 | 17.0 | 0.302 |
| Eating habits |  |  |  |  |  |
| Very regular | 92 | 62.6 | 116 | 78.9 | 0.002 |
| Not very regular | 55 | 37.4 | 31 | 21.1 | 0.002 |
| Self-perceived health |  |  |  |  |  |
| Very good | 11 | 7.5 | 22 | 15.0 | 0.042 |
| Good | 62 | 42.2 | 63 | 42.8 | 0.906 |
| Ordinary | 56 | 38.1 | 55 | 37.4 | 0.904 |
| Not good | 18 | 12.2 | 7 | 4.8 | 0.019 |
